# The Role of The Rostral Ventromedial Medulla in Stress Responses

**DOI:** 10.3390/brainsci13050776

**Published:** 2023-05-09

**Authors:** Marco Pagliusi, Felipe V. Gomes

**Affiliations:** Department of Pharmacology, Ribeirão Preto Medical School, University of São Paulo, Ribeirão Preto 14015-069, SP, Brazil; mpagliusi@usp.br

**Keywords:** stress-induced analgesia, stress-induced hyperalgesia, opioid system, endocannabinoid system, pain, chronic pain, pain chronification

## Abstract

The rostral ventromedial medulla (RVM) is a brainstem structure critical for the descending pain modulation system involved in both pain facilitation and inhibition through its projection to the spinal cord. Since the RVM is well connected with pain- and stress-engaged brain structures, such as the anterior cingulate cortex, nucleus accumbens, and amygdala, its involvement in stress responses has become a matter of great interest. While chronic stress has been proposed as a trigger of pain chronification and related psychiatric comorbidities due to maladaptive stress responses, acute stress triggers analgesia and other adaptative responses. Here we reviewed and highlighted the critical role of the RVM in stress responses, mainly in acute stress-induced analgesia (SIA) and chronic stress-induced hyperalgesia (SIH), providing insights into pain chronification processes and comorbidity between chronic pain and psychiatric disorders.

## 1. Introduction

The rostral ventromedial medulla (RVM) is a key brainstem structure that plays a critical role as a relay for facilitating and inhibiting pain as part of the descending pain modulation system. RVM anatomical delimitation comprises the nucleus raphe magnus and the adjacent reticular formation at the level of the facial nucleus and ventral to the gigantocellular reticular nucleus [1,2]. This descending pain system operates as an endogenous control of pain, being recruited following painful stimuli perception alongside many other brain regions, including the primary and secondary somatosensory cortices, anterior cingulate cortex (ACC), prefrontal cortex (PFC), insula, amygdala, nucleus accumbens (NAc), ventral tegmental area (VTA), and periaqueductal grey (PAG) [3,4]. Each of these brain regions is hypothesized to be involved in one of the core aspects of pain, defined as the sensorial-discriminative and affective-motivational dimensions [3,4,5]. The first encodes sensorial features of pain, including location, duration, and intensity, which are presumed to be processed by the primary and secondary somatosensory cortices and the insula [5,6]. The latter encodes emotional and contextual aspects of pain, such as suffering and contextual avoidance, and is processed by the PFC, ACC, amygdala, VTA, and NAc [5,6].

**Figure 1:**
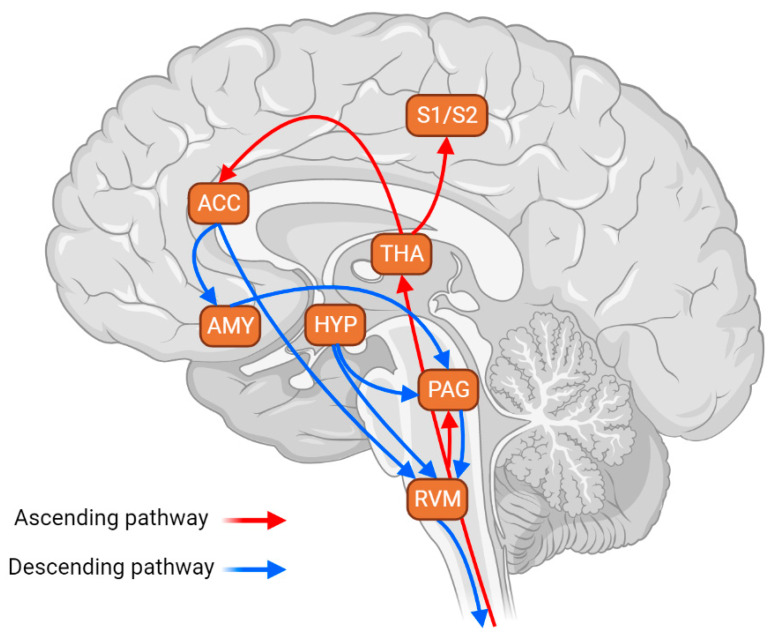
Scheme of the major connections projecting, directly or indirectly, to the RVM. This circuitry is critical in modulating the descending and ascending pain pathways. Arrows in red indicate the ascending pain pathway that conveys nociceptive signals from the spinal cord to supra-spinal structures responsible for pain processing—these ascending signals relay in the periaqueductal gray (PAG) and thalamus (THA). The latter sends projections to the primary and secondary somatosensorial cortices (S1 and S2) and anterior cingulate cortex (ACC). Arrows in blue indicate the descending pain pathway, which modulates the ascending information by facilitating or inhibiting its signals. The key structure of the descending pain modulation system is the rostral ventromedial medulla (RVM), which receives major projections from the PAG. The PAG-RVM pathway is one of the best-studied circuits in the descending pain modulation system. Its activation facilitates and inhibits pain. Both structures, the PAG and RVM, receive inputs from the hypothalamus (HYP), a well-known structure implicated in the modulation of stress responses. The PAG also receives inputs from the amygdala (AMY), which is also implicated in stress responses. Finally, the ACC sends projections to the AMY, indirectly influencing the descending pain modulation, and to the RVM, directly influencing the descending pain modulation output. Hence, the RVM maintains, directly or indirectly, neuronal connections with most pain-engaged structures, providing a mechanism in which cortical and subcortical structures influence nociceptive input and pain perception. Therefore, this circuitry is a critical neuroanatomical substrate for stress-induced alterations in nociceptive sensitivity.

The RVM maintains neuronal connections with most pain-engaged structures, providing a mechanism through which cortical and subcortical structures influence nociceptive input and pain perception [6,7,8,9]. Notably, the RVM connectivity underlies the placebo analgesia, which also highlights the great relevance of the RVM in pain control as it can impact both sensorial-discriminative and affective-motivational aspects of pain [10]. A schematic representation of the major connections that directly or indirectly project to the RVM and play a role in the modulation of the descending and ascending pain pathways is shown in Figure 1. One of the most well-investigated projections for the RVM arises from the PAG. When endogenously or exogenously (e.g., through chemogenetics) stimulated, PAG-RVM projections exert analgesia by facilitating the descending inhibition of pain [9]. Inversely, projections from the ACC to the RVM have been described as an enhancer of the descending facilitation of pain. In addition, the overactivation of ACC-RVM projections is thought to be involved in pain chronification [9,10]. Based on its key role in modulating pain responses, the RVM has been implicated as an important brain region in the switch from acute to chronic pain [9,11,12]. Several studies suggest that pain chronification relies on an imbalance between the facilitatory and inhibitory roles of the RVM in controlling nociceptive input [6,9,11,12]. In this context, it has been proposed that the basal sensorial processing threshold is maintained by a fine balance between the descending pain facilitatory and inhibitory systems, which can be disturbed by illness, lesions, inflammation, or even exposure to psychological stressors [6,13,14].

Regarding the pain facilitatory and inhibitory roles of the RVM, the contrasting involvement of its different cell types during pain processing is remarkable. While there are neurons in the RVM that facilitate pain and are called *on-cells*, some neurons inhibit pain and are called *off-cells* [1,12,15]. The imbalance between descending pain facilitatory and inhibitory systems in the establishment of chronic pain is proposed to be mediated by dissonance in activating *on-* and *off-cells* [6,12]. Both cell types are simultaneously activated in the early stages of pain. However, during pain chronification, *on-cells* show a stronger signal strength, overcoming the *off-cells* to promote chronic pain [12]. In addition to *on-* and *off-cells*, there is another group of cells in the RVM that is neither pro- nor anti-nociceptive, and they are called *neutral-cells*. The role of *neutral-cells* in descending pain control is still unclear, but it is thought that they may change their activity and be recruited as *on-* or *off-cells* during pain chronification [15,16,17].

Based on the critical role of the RVM in pain chronification and its privileged neuroanatomical connectivity, its involvement in stress responses, mainly stress-induced hyperalgesia and allodynia, has been a matter of interest. While hyperalgesia is defined as increased pain sensitivity, allodynia is defined as pain in response to a non-nociceptive stimulus [18]. Several pieces of evidence point to chronic stress as a trigger for pain chronification and related psychiatric comorbidities (e.g., depression and anxiety), which are associated with maladaptive responses to stress [19,20].

Epidemiological studies indicate that stressful life events can act as major risk factors for the development of depression and chronic pain [21]. Additionally, several animal models for both disorders employ chronic stress stimuli, such as repeated restraint stress and chronic social defeat stress [22,23,24,25]. For example, it was shown that ten days of social defeat stress induces long-lasting allodynia and hyperalgesia along with behavioral impairments related to depression, such as social avoidance and decreased sucrose preference [24,26]. On the other hand, acute stressors trigger analgesia [27]. This response is part of a natural repertory engaged during stressful situations for individual surveillance and homeostasis [27,28,29]. Accordingly, the terms acute stress-induced analgesia (SIA) and chronic stress-induced hyperalgesia (SIH) are commonly used in the context of stress and nociceptive sensibility. Not surprisingly, due to its role in nociceptive processes, the involvement of the RVM in stress responses relies on both conditions.

Most pain-engaged structures that directly or indirectly maintain neuronal connections within the RVM, such as the PFC, ACC, amygdala, and NAc, are recruited during exposure to stressors or modify stress responses [20,29,30,31]. Therefore, understanding the role of the RVM in modulating stress responses may provide insights into pain chronification processes and comorbidity between chronic pain and psychiatric disorders, mainly those related to exposure to stressful events (e.g., depression). Here, we reviewed and discussed key aspects of RVM engagement during stress and how it can be implicated in deleterious stress outcomes, shedding light on the molecular and physiological mechanisms behind them.

## 2. RVM Mediates Stress Response through the Endocannabinoid and Opioid Systems

The RVM and the pathways relaying on it are well-provided with opioid and cannabinoid receptors, highlighting their key role in the analgesic action of both endogenous systems and exogenous drugs [32,33,34,35]. One of the main sources of endogenous opioid release into the RVM is the hypothalamus [36]. Whereas RVM *on-cells* express µ-opioid receptors (MOR), *off-cells* express kappa-opioid receptors (KOR) [36]. The latter is disinhibited by opioid administration, mediating the analgesic effect of opioids in an RVM-dependent manner [37,38]. Moreover, the RVM is also enriched with the cannabinoid type-1 receptor (CB_1_), which produces analgesia when activated [39,40]. Based on this, one could expect the involvement of the endocannabinoid and opioid systems in stress responses mediated by the RVM, mainly in nociceptive alterations, which have been the most common RVM-related stress outcomes investigated.

A critical role of the RVM endocannabinoid and opioid systems in acute stress-induced analgesia (SIA) has been extensively described. Regarding the endocannabinoid system, a potentiation of the neurotransmission mediated by endocannabinoids within the RVM leads to an enhancement in SIA. The inhibition of the fatty-acid amide hydrolase (FAAH), an enzyme that degrades the endocannabinoid anandamide (AEA), in the RVM increases SIA [41]. Blocking FAAH is an important strategy to potentiate endocannabinoid neurotransmission since it increases AEA levels. This positive effect was blocked by the intra-RVM infusion of rimonabant, a CB1 receptor antagonist, indicating that SIA depends on the activation of local CB1 receptors [41]. Similarly, using Wistar-Kyoto rats, a genotype-dependent stress-hyperresponsive inbred strain, it was shown that this strain had decreased levels of AEA and the other major endocannabinoid 2-arachidonoylglycerol (2-AG) in the RVM and a great nociceptive response following formalin injection in the hind paw compared to the control rat, which was attenuated by the pharmacological inhibition of FAAH in the RVM [42]. In addition, in Wistar-Kyoto rats, the pharmacological blockade of CB1 receptors in the RVM potentiated hyperalgesia [42].

Exposure to acute stressors increases the release of 2-AG and AEA into the PAG, one of the most important regions projecting to the RVM, eliciting SIA [43]. This PAG-dependent SIA can be prevented by the local antagonism of CB1 receptors. On the contrary, local FAAH inhibition enhances it [43]. Additionally, stress enhances glutamatergic signaling into the PAG, leading to local endocannabinoid production and signaling to an analgesic state [44]. The cannabinoid-dependent SIA is also accompanied by an increase in 2-AG but not AEA at the spinal cord level, the main RVM projection target [45]. However, the antagonism of CB1 receptors in this region did not block SIA, suggesting that the endocannabinoids at the spinal level may be involved in, but do not directly mediate, SIA [45]. Together, these results highlight a possible mechanism for stress-induced nociceptive alterations and cannabinoid-based chronic pain treatments, which may involve the RVM [35,41,43,46,47]. It is even more relevant considering the dramatic increase in deaths caused by opioid overdoses lately [46].

In addition to its effects through the endocannabinoid system, the RVM also impacts SIA via the endogenous opioid system. It was found that SIA is mediated by RVM µ-opioid receptors since the intra-RVM injection of CTAP, a µ-opioid receptor antagonist, or antisense to downregulate their expression, blocked SIA emergence. On the contrary, RVM κ- and δ-opioid receptors do not modulate SIA [48,49]. The pro-SIA effect mediated by the antagonism of RVM µ-opioid receptors was diminished by the activation of local κ-opioid receptors [50,51]. It indicates a possible mechanism for pain chronification establishment since chronic stress can increase the neurotransmission mediated by κ-opioid receptors during the switch from SIA to stress-induced hyperalgesia (SIH). This RVM-specific mechanism contrasts with a study showing that systemic κ-opioid antagonism attenuates analgesia induced by social defeat stress, an effect probably mediated by spinal cord neurons expressing κ-opioid receptor [52,53]. In addition, a recent study demonstrated that κ-opioid receptor-expressing neurons in the RVM, mainly *off-cells* (pro-analgesia), are required for SIA expression through a spinal cord-targeted mechanism, characterization of an important RVM descendent pathway opioid-responsive capable of influencing the stress response [16,54].

Apart from the implication of the RVM in acute SIA, it was shown that RVM is involved in chronic SIH [55]. Specifically, selective ablation of the RVM µ-opioid receptor-expressing neurons attenuated SIH. It may indicate that these receptors are involved in SIA and SIH by acting differentially through *on-* or *off-cells* in the RVM [48,49,55]. µ-opioid receptor agonists inhibit *on-cells* (pro-nociceptive) and activate *off-cells* (pro-analgesia) [37,56,57]. Since the activation of *off-cells* is mediated by presynaptic inhibition of GABAergic terminals, the ablation of µ-opioid receptor-expressing neurons in the RVM would negatively affect the pro-nociceptive pathway [33,37,56]. Additionally, the anti-opioid neuropeptide cholecystokinin (CCK), an anxiogenic and stress-related agent, activates *on-cells* and triggers hyperalgesia when injected intra-RVM [57,58].

## 3. Involvement of RVM-Targeting Pathways in Stress Response

As mentioned above, the RVM is a relay for the descending pain modulation system. Therefore, several important structures in the brain send projections to it, many of which remain to be further investigated to provide insights into their role in stress responses and the descending pain modulation system. One of these projections, directly and indirectly, originates in the central nucleus of the amygdala (CeA), a well-known stress-related nucleus. The injection of α2-adrenergic receptor agonist into the CeA results in hypoalgesia, evidencing a possible role for this nucleus in SIA [59]. Interestingly, this CeA-induced hypoalgesia was not prevented by RVM chemical inhibition, systemic injections of naloxone (an opioid receptor antagonist), or rimonabant (a CB1 receptor antagonist), evidencing a possible non-opioid-endocannabinoid-dependent mechanism for SIA [59]. The CeA-induced hypoalgesia seems to be mediated by adrenergic neurotransmission at the spinal cord level, given that the intrathecal injection of an α2-adrenergic receptor antagonist blocked this hypoalgesia [59]. Further, the basolateral amygdala (BLA), which is also involved in stress responses, can influence the RVM through its indirect connections through the PAG. It was demonstrated that opioid stimulation of the BLA triggers antinociception in a PAG-RVM-dependent manner. Additionally, different amygdala nuclei might influence the RVM *on-* and *off-cells* differently, given that only the basolateral, medial, and cortical nuclei, but not the central, medial lateral, and dorsal lateral nuclei, seem to modulate RVM activity [60,61].

Another important brain region implicated in stress responses that directly and indirectly projects to the RVM is the dorsomedial nucleus of the hypothalamus (DMH). The DMH is critical in controlling neuroendocrine, cardiovascular, and thermogenic responses to stress [62,63]. The stimulation of the DMH, in addition to inducing stress-related outcomes evoking increased heart rate and hyperthermia, induced hyperalgesia [64]. Moreover, the activation of the DMH leads to the activation of the RVM *on-cells* and suppression of *off-cells*, a mechanism that precipitates hyperalgesia [64]. Further, blocking the activation of *on-cells* prevented the hyperalgesia induced by DMH activation [64]. Corroborating this study, it was also shown that after chronic exposure to stress, the DMH releases the anxiogenic and stress-related agent CCK in the RVM, which will act as a pro-nociceptive molecule by activating *on-cells* [57,65].

Interestingly, SIH caused by prolonged stress stimulus is accompanied by increases in the levels of CCK receptors in the RVM and is blocked by the infusion of CCK receptor antagonists intra-RVM [66]. It is corroborated by a study in rats showing that social defeat stress outcomes, including chronic hyperalgesia and anxiety-like behaviors, were attenuated by intra-RVM injection of a CCK antagonist [67]. Together, these results suggest a possible mechanism for SIH and a target for new studies to understand the pain chronification process and its comorbidity with psychiatric disorders.

## 4. Other Mechanisms Implicating the RVM in Stress Response

Most of the descending projections from the RVM to the dorsal horn of the spinal cord are serotonergic neurons [6]. In this context, SIH is marked by higher levels of the phosphorylated extracellular signal-regulated kinase (pERK) along with higher levels of tryptophan hydroxylase (TPH) in the RVM serotonergic neurons, indicating an increase in the descending serotonergic neurotransmission [68]. On the contrary, a rodent model relevant to post-traumatic stress disorder presents SIH in addition to a decrease in serotonergic neurons in the dorsal raphe nucleus (DRN), which is not part of the RVM anatomical delimitation [69]. These results indicate that stress may have differential impacts on distinct populations of serotonergic neurons, potentially leading to different outcomes. These RVM serotonergic neurons are also implicated in SIA. Intra-RVM injection of serotonergic receptor antagonists potentiates SIA, while glutamate NMDA receptor antagonists have no effect [70]. This result highlights the critical role of the RVM serotonergic system in SIA.

Further, in addition to RVM descending serotonergic projections and GABAergic projections from the RVM synapse onto spinal enkephalinergic interneurons [71], this synapse facilitates nociception and is implicated in SIA and SIH since these GABAergic neurons are activated during SIA and inhibited during SIH [71]. These results uncovered a still unknown GABAergic RVM-descending projection engaged during stress and suggest a possible target to deepen into the mechanism by which stress exposure can lead to pain chronification. Another potential target implicated in this mechanism is the brain-derived neurotrophic factor signaling pathway. It was shown that maternal separation, a model for depressive and anxiety-like behaviors, induces hyperalgesia. This increased pain response was associated with increased levels of the tyrosine kinase receptor B in the RVM but without changes in its ligand, BDNF [72].

Diffuse noxious inhibitory control (DNIC) is another widely used paradigm that is relevant in this context. It shares great conceptual similarities with SIA, and, as expected, its mechanism depends on the activity of the RVM [27]. DNIC is a very powerful and long-lasting descending inhibitor of pain following a noxious stimulus [27], being characterized when a response from a painful stimulus is inhibited by another noxious stimulus applied to any part of the body, distinct from its excitatory receptive fields. Interestingly, it was shown that the RVM *off-cells* might be implicated in the DNIC processing since inhibiting these cells through GABA_A_ agonism attenuates the DNIC-induced analgesia with no participation of *on-cells* [73]. The conditioned pain modulation (CPM) paradigm is used to assess the DNIC in humans, in which an experimenter applies a painful stimulus in a specific body area that reduces the pain from a standardized noxious stimulus applied at a different body area. It is known that chronic pain and depressive patients show a less efficient CPM response, pointing to the RVM as a relevant brain structure for these disorders that are often manifested concomitantly [74,75].

## 5. Concluding Remarks

**Figure 2:**
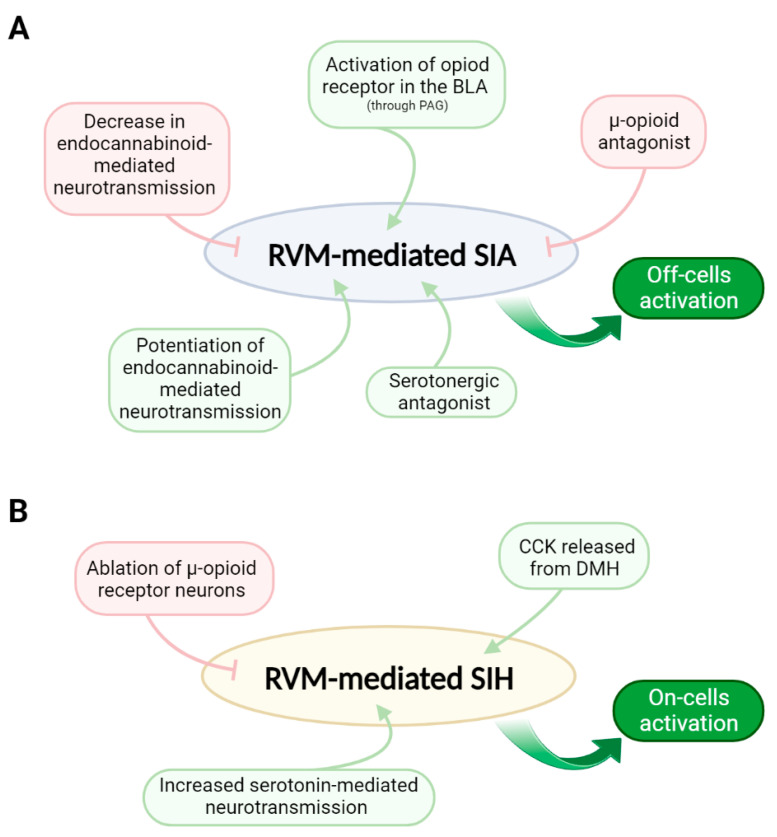
RVM mechanisms involved in the generation of acute stress-induced analgesia (SIA) and chronic stress-induced hyperalgesia (SIH). (**A**) In general, SIA involves the activation of RVM *off-cells*, which triggers a decrease in the nociceptive response, generating analgesia. Decreases in endocannabinoid-mediated neurotransmission and the antagonism of µ-opioid receptors in the RVM block SIA. On the other hand, the antagonism of serotonergic receptors, the activation of opioid receptors in the BLA, and the potentiation of endocannabinoid-mediated neurotransmission in the RVM facilitate SIA. (**B**) SIH involves the activation of RVM *on-cells*, which triggers an increase in the nociceptive response, i.e., hyperalgesia and allodynia. The ablation of µ-opioid receptor-expressing neurons in the RVM blocks SIH, while CCK released from DMH and increased serotonin-mediated neurotransmission in the RVM potentiate SIH. Green arrows indicate activation, and red lines indicate inhibition. BLA: basolateral amygdala; PAG: periaqueductal grey; CCK: cholecystokinin, an anxiogenic and stress-related agent; DMH: dorsomedial nucleus of the hypothalamus.

Here, we reviewed the critical role of the RVM in modulating stress responses, mainly SIA and SIH (Figure 2). The RVM impacts the nociceptive signal at the spinal cord level through serotonergic and GABAergic projections, which can be directly or indirectly impacted by the endocannabinoid and opioid systems, besides neuropeptides (e.g., CCK) and other molecules released by several supra-RVM brain structures. Most studies aiming to investigate the RVM impact on stress outcomes mainly focused on the nociceptive response (both SIA and SIH), i.e., did not further the analysis on other behavioral impairments triggered by stress, such as anhedonia. Although there might be different mechanisms for SIA and SIH, the first seems to be mediated by the activation of RVM *off-cells*. In contrast, the latter appears to be mediated by the recruitment of the RVM *on-cells*. Here we gathered evidence and spotlighted the RVM as a possible anatomical substrate for the great comorbidity between chronic pain and psychiatric disorders, such as anxiety and depression, which are commonly associated with chronic stress. However, despite the involvement of the RVM in nociceptive response has been deeply investigated, the possible involvement of the RVM in more general stress outcomes still needs to be better assessed and should be a potential target for future studies.

## Data Availability

No new data were created or analyzed in this study. Data sharing is not applicable to this article.

## References

[1] Fields H.L., Bry J., Hentall I., Zorman G. (1983). The Activity of Neurons in the Rostral Medulla of the Rat during Withdrawal from Noxious Heat. J. Neurosci..

[2] Fields H.L., Heinricher M.M. (1985). Anatomy and Physiology of a Nociceptive Modulatory System. Philos. Trans. R. Soc. Lond. B. Biol. Sci..

[3] Denk F., McMahon S.B., Tracey I. (2014). Pain Vulnerability: A Neurobiological Perspective. Nat. Neurosci..

[4] Bushnell M.C., Čeko M., Low L.A. (2013). Cognitive and Emotional Control of Pain and Its Disruption in Chronic Pain. Nat. Rev. Neurosci..

[5] Auvray M., Myin E., Spence C. (2010). The Sensory-Discriminative and Affective-Motivational Aspects of Pain. Neurosci. Biobehav. Rev..

[6] Ossipov M.H., Morimura K., Porreca F. (2014). Descending Pain Modulation and Chronification of Pain. Curr. Opin. Support. Palliat. Care.

[7] Fields H. (2004). State-Dependent Opioid Control of Pain. Nat. Rev. Neurosci..

[8] Basbaum A.I., Bautista D.M., Scherrer G., Julius D. (2009). Cellular and Molecular Mechanisms of Pain. Cell.

[9] Zhuo M. (2017). Descending Facilitation: From Basic Science to the Treatment of Chronic Pain. Mol. Pain.

[10] De Felice M., Ossipov M.H. (2016). Cortical and Subcortical Modulation of Pain. Pain Manag..

[11] De Felice M., Sanoja R., Wang R., Vera-Portocarrero L., Oyarzo J., King T., Ossipov M.H., Vanderah T.W., Lai J., Dussor G.O. (2011). Engagement of Descending Inhibition from the Rostral Ventromedial Medulla Protects against Chronic Neuropathic Pain. Pain.

[12] Weiwei Y., Wendi F., Mengru C., Tuo Y., Chen G. (2021). The Cellular Mechanism by Which the Rostral Ventromedial Medulla Acts on the Spinal Cord during Chronic Pain. Rev. Neurosci..

[13] Ferdousi M., Finn D.P. (2018). Stress-Induced Modulation of Pain: Role of the Endogenous Opioid System.

[14] Crofford L.J. (2015). Chronic Pain: Where the Body Meets the Brain. Trans. Am. Clin. Climatol. Assoc..

[15] Heinricher M., Tavares I., Leith J.L., Lumb B.M. (2010). Descending Control of Nociception. Brain Res. Rev..

[16] Winkler C.W., Hermes S.M., Chavkin C.I., Drake C.T., Morrison S.F., Aicher S.A. (2006). Kappa Opioid Receptor (KOR) and GAD67 Immunoreactivity Are Found in OFF and NEUTRAL Cells in the Rostral Ventromedial Medulla. J. Neurophysiol..

[17] Miki K., Zhou Q.Q., Guo W., Guan Y., Terayama R., Dubner R., Ren K. (2002). Changes in Gene Expression and Neuronal Phenotype in Brain Stem Pain Modulatory Circuitry after Inflammation. J. Neurophysiol..

[18] Sandkühler J. (2009). Models and Mechanisms of Hyperalgesia and Allodynia. Physiol. Rev..

[19] Dean J., Keshavan M. (2017). The Neurobiology of Depression: An Integrated View. Asian J. Psychiatry.

[20] Sheng J., Liu S., Wang Y., Cui R., Zhang X. (2017). The Link between Depression and Chronic Pain: Neural Mechanisms in the Brain. Neural Plast..

[21] Maletic V., Raison C.L. (2009). Neurobiology of Depression, Fibromyalgia and Neuropathic Pain. Front. Biosci. (Landmark Ed.).

[22] Imbe H., Iwai-Liao Y., Senba E. (2006). Stress-Induced Hyperalgesia: Animal Models and Putative Mechanisms. Front. Biosci..

[23] Pagliusi M.O.F., Bonet I.J.M., Dias E.V., Vieira A.S., Tambeli C.H., Parada C.A., Sartori C.R. (2018). Social Defeat Stress Induces Hyperalgesia and Increases Truncated BDNF Isoforms in the Nucleus Accumbens Regardless of the Depressive-like Behavior Induction in Mice. Eur. J. Neurosci..

[24] Pagliusi M., Bonet I.J.M., Brandão A.F., Magalhães S.F., Tambeli C.H., Parada C.A., Sartori C.R. (2020). Therapeutic and Preventive Effect of Voluntary Running Wheel Exercise on Social Defeat Stress (SDS)-Induced Depressive-like Behavior and Chronic Pain in Mice. Neuroscience.

[25] Jennings E.M., Okine B.N., Roche M., Finn D.P. (2014). Stress-Induced Hyperalgesia. Prog. Neurobiol..

[26] Piardi L., Pagliusi M., Bonet I., Brandão A., Magalhães S., Zanelatto F., Tambeli C., Parada C., Sartori C. (2020). Social Stress as a Trigger for Depressive-like Behavior and Persistent Hyperalgesia in Mice: Study of the Comorbidity between Depression and Chronic Pain. J. Affect. Disord..

[27] Butler R.K., Finn D.P. (2009). Stress-Induced Analgesia. Prog. Neurobiol..

[28] Li X.-M., Meng J., Li L.-T., Guo T., Yang L.-K., Shi Q.-X., Li X.-B., Chen Y., Yang Q., Zhao J.-N. (2017). Effect of ZBD-2 on Chronic Pain, Depressive-like Behaviors, and Recovery of Motor Function Following Spinal Cord Injury in Mice. Behav. Brain Res..

[29] Nestler E.J., Waxman S.G. (2020). Resilience to Stress and Resilience to Pain: Lessons from Molecular Neurobiology and Genetics. Trends Mol. Med..

[30] Vialou V., Robison A.J., Laplant Q.C., Covington H.E., Dietz D.M., Ohnishi Y.N., Mouzon E., Rush A.J., Watts E.L., Wallace D.L. (2010). ΔfosB in Brain Reward Circuits Mediates Resilience to Stress and Antidepressant Responses. Nat. Neurosci..

[31] Laine M.A., Sokolowska E., Dudek M., Callan S.A., Hyytiä P., Hovatta I. (2017). Brain Activation Induced by Chronic Psychosocial Stress in Mice. Sci. Rep..

[32] Hathway G.J., Vega-Avelaira D., Fitzgerald M. (2012). A Critical Period in the Supraspinal Control of Pain: Opioid-Dependent Changes in Brainstem Rostroventral Medulla Function in Preadolescence. Pain.

[33] Fields H.L., Vanegas H., Hentall I.D., Zorman G. (1983). Evidence That Disinhibition of Brain Stem Neurones Contributes to Morphine Analgesia. Nature.

[34] Vanegas H., Vazquez E., Tortorici V. (2010). NSAIDS, Opioids, Cannabinoids and the Control of Pain by the Central Nervous System. Pharmaceuticals.

[35] Meng I.D., Manning B.H., Martin W.J., Fields H.L. (1998). An Analgesia Circuit Activated by Cannabinoids. Nature.

[36] Bagley E.E., Ingram S.L. (2020). Endogenous Opioid Peptides in the Descending Pain Modulatory Circuit. Neuropharmacology.

[37] Heinricher M.M., Morgan M.M., Tortorici V., Fields H.L. (1994). Disinhibition of Off-Cells and Antinociception Produced by an Opioid Action within the Rostral Ventromedial Medulla. Neuroscience.

[38] Mitchell J.M., Lowe D., Fields H.L. (1998). The Contribution of the Rostral Ventromedial Medulla to the Antinociceptive Effects of Systemic Morphine in Restrained and Unrestrained Rats. Neuroscience.

[39] Corcoran L., Roche M., Finn D.P. (2015). The Role of the Brain’s Endocannabinoid System in Pain and Its Modulation by Stress.

[40] Palazzo E., Luongo L., de Novellis V., Rossi F., Maione S. (2010). The Role of Cannabinoid Receptors in the Descending Modulation of Pain. Pharmaceuticals.

[41] Suplita R.L., Farthing J.N., Gutierrez T., Hohmann A.G. (2005). Inhibition of Fatty-Acid Amide Hydrolase Enhances Cannabinoid Stress-Induced Analgesia: Sites of Action in the Dorsolateral Periaqueductal Gray and Rostral Ventromedial Medulla. Neuropharmacology.

[42] Rea K., Olango W.M., Okine B.N., Madasu M.K., McGuire I.C., Coyle K., Harhen B., Roche M., Finn D.P. (2014). Impaired Endocannabinoid Signalling in the Rostral Ventromedial Medulla Underpins Genotype-Dependent Hyper-Responsivity to Noxious Stimuli. Pain.

[43] Hohmann A.G., Suplita R.L., Bolton N.M., Neely M.H., Fegley D., Mangieri R., Krey J.F., Walker J.M., Holmes P.V., Crystal J.D. (2005). An Endocannabinoid Mechanism for Stress-Induced Analgesia. Nature.

[44] Drew G.M., Mitchell V.A., Vaughan C.W. (2008). Glutamate Spillover Modulates GABAergic Synaptic Transmission in the Rat Midbrain Periaqueductal Grey via Metabotropic Glutamate Receptors and Endocannabinoid Signaling. J. Neurosci..

[45] Suplita R.L., Gutierrez T., Fegley D., Piomelli D., Hohmann A.G. (2006). Endocannabinoids at the Spinal Level Regulate, but Do Not Mediate, Nonopioid Stress-Induced Analgesia. Neuropharmacology.

[46] Urits I., Gress K., Charipova K., Habib K., Lee D., Lee C., Jung J.W., Kassem H., Cornett E., Paladini A. (2020). Use of Cannabidiol (CBD) for the Treatment of Chronic Pain. Best Pract. Res. Clin. Anaesthesiol..

[47] Martin W.J., Tsou K., Walker J.M. (1998). Cannabinoid Receptor-Mediated Inhibition of the Rat Tail-Flick Reflex after Microinjection into the Rostral Ventromedial Medulla. Neurosci. Lett..

[48] Foo H., Helmstetter F.J. (2000). Expression of Antinociception in Response to a Signal for Shock Is Blocked after Selective Downregulation of μ-Opioid Receptors in the Rostral Ventromedial Medulla. Mol. Brain Res..

[49] Foo H., Helmstetter F.J. (1999). Hypoalgesia Elicited by a Conditioned Stimulus Is Blocked by a μ, but Not a δ or a κ, Opioid Antagonist Injected into the Rostral Ventromedial Medulla. Pain.

[50] Foo H., Helmstetter F.J. (2000). Activation of Kappa Opioid Receptors in the Rostral Ventromedial Medulla Blocks Stress-Induced Antinociception. Neuroreport.

[51] Meng I.D., Johansen J.P., Harasawa I., Fields H.L. (2005). Kappa Opioids Inhibit Physiologically Identified Medullary Pain Modulating Neurons and Reduce Morphine Antinociception. J. Neurophysiol..

[52] McLaughlin J.P., Li S., Valdez J., Chavkin T.A., Chavkin C. (2006). Social Defeat Stress-Induced Behavioral Responses Are Mediated by the Endogenous Kappa Opioid System. Neuropsychopharmacology.

[53] Otsu Y., Aubrey K.R. (2022). Kappa Opioids Inhibit the GABA/Glycine Terminals of Rostral Ventromedial Medulla Projections in the Superficial Dorsal Horn of the Spinal Cord. J. Physiol..

[54] Nguyen E., Smith K.M., Cramer N., Holland R.A., Bleimeister I.H., Flores-Felix K., Silberberg H., Keller A., Le Pichon C.E., Ross S.E. (2022). Medullary Kappa-Opioid Receptor Neurons Inhibit Pain and Itch through a Descending Circuit. Brain.

[55] Reynolds J., Bilsky E.J., Meng I.D. (2011). Selective Ablation of Mu-Opioid Receptor Expressing Neurons in the Rostral Ventromedial Medulla Attenuates Stress-Induced Mechanical Hypersensitivity. Life Sci..

[56] Heinricher M.M., Haws C.M., Fields H.L. (1991). Evidence for GABA-Mediated Control of Putative Nociceptive Modulating Neurons in the Rostral Ventromedial Medulla: Iontophoresis of Bicuculline Eliminates the off-Cell Pause. Somatosens. Mot. Res..

[57] Heinricher M.M., Neubert M.J. (2004). Neural Basis for the Hyperalgesic Action of Cholecystokinin in the Rostral Ventromedial Medulla. J. Neurophysiol..

[58] Friedrich A.E., Gebhart G.F. (2003). Modulation of Visceral Hyperalgesia by Morphine and Cholecystokinin from the Rat Rostroventral Medial Medulla. Pain.

[59] Maire J.J., Close L.N., Heinricher M.M., Selden N.R. (2016). Distinct Pathways for Norepinephrine- and Opioid-Triggered Antinociception from the Amygdala. Eur. J. Pain.

[60] Helmstetter F.J., Tershner S.A., Poore L.H., Bellgowan P.S.F. (1998). Antinociception Following Opioid Stimulation of the Basolateral Amygdala Is Expressed through the Periaqueductal Gray and Rostral Ventromedial Medulla. Brain Res..

[61] McGaraughty S., Heinricher M.M. (2002). Microinjection of Morphine into Various Amygdaloid Nuclei Differentially Affects Nociceptive Responsiveness and RVM Neuronal Activity. Pain.

[62] Lamotte G., Shouman K., Benarroch E.E. (2021). Stress and Central Autonomic Network. Auton. Neurosci..

[63] Myers B., Mark Dolgas C., Kasckow J., Cullinan W.E., Herman J.P. (2014). Central Stress-Integrative Circuits: Forebrain Glutamatergic and GABAergic Projections to the Dorsomedial Hypothalamus, Medial Preoptic Area, and Bed Nucleus of the Stria Terminalis. Brain Struct. Funct..

[64] Martenson M.E., Cetas J.S., Heinricher M.M. (2009). A Possible Neural Basis for Stress-Induced Hyperalgesia. Pain.

[65] Wagner K.M., Roeder Z., DesRochers K., Buhler A.V., Heinricher M.M., Cleary D.R. (2013). The Dorsomedial Hypothalamus Mediates Stress-Induced Hyperalgesia and Is the Source of the Pronociceptive Peptide Cholecystokinin in the Rostral Ventromedial Medulla. Neuroscience.

[66] Jiang M., Bo J., Lei Y., Hu F., Xia Z., Liu Y., Lu C., Sun Y., Hou B., Ni K. (2019). Anxiety-Induced Hyperalgesia in Female Rats Is Mediated by Cholecystokinin 2 Receptor in Rostral Ventromedial Medulla and Spinal 5-Hydroxytryptamine 2B Receptor. J. Pain Res..

[67] Rivat C., Becker C., Blugeot A., Zeau B., Mauborgne A., Pohl M., Benoliel J.J. (2010). Chronic Stress Induces Transient Spinal Neuroinflammation, Triggering Sensory Hypersensitivity and Long-Lasting Anxiety-Induced Hyperalgesia. Pain.

[68] Imbe H., Murakami S., Okamoto K., Iwai-Liao Y., Senba E. (2004). The Effects of Acute and Chronic Restraint Stress on Activation of ERK in the Rostral Ventromedial Medulla and Locus Coeruleus. Pain.

[69] Vieira J.S., de Souza G.R., Kalil-Cutti B., Giusti-Paiva A., Vilela F.C. (2021). Post-Traumatic Stress Disorder Increases Pain Sensitivity by Reducing Descending Noradrenergic and Serotoninergic Modulation. Behav. Brain Res..

[70] Hopkins E., Spinella M., Pavlovic Z.W., Bodnar R.J. (1998). Alterations in Swim Stress-Induced Analgesia and Hypothermia Following Serotonergic or NMDA Antagonists in the Rostral Ventromedial Medulla of Rats. Physiol. Behav..

[71] François A., Low S.A., Sypek E.I., Christensen A.J., Sotoudeh C., Beier K.T., Ramakrishnan C., Ritola K.D., Sharif-Naeini R., Deisseroth K. (2017). A Brainstem-Spinal Cord Inhibitory Circuit for Mechanical Pain Modulation by GABA and Enkephalins. Neuron.

[72] Chung E.K.Y., Bian Z.X., Xu H.X., Sung J.J.Y. (2009). Neonatal Maternal Separation Increases Brain-Derived Neurotrophic Factor and Tyrosine Kinase Receptor B Expression in the Descending Pain Modulatory System. NeuroSignals.

[73] Chebbi R., Boyer N., Monconduit L., Artola A., Luccarini P., Dallel R. (2014). The Nucleus Raphe Magnus OFF-Cells Are Involved in Diffuse Noxious Inhibitory Controls. Exp. Neurol..

[74] Yarnitsky D. (2015). Role of Endogenous Pain Modulation in Chronic Pain Mechanisms and Treatment. Pain.

[75] Chen Q.L., Heinricher M.M. (2019). Descending Control Mechanisms and Chronic Pain. Curr. Rheumatol. Rep..

